# Genomic profile of an IDH−wild−type glioblastoma diagnosed following TNF−α inhibitor therapy: a molecular case study

**DOI:** 10.3389/fonc.2026.1788983

**Published:** 2026-04-28

**Authors:** Suryanarayan Mohapatra, Natarajan Ganesan

**Affiliations:** 1 Internal Medicine, Kaiser Permanente - Mid-Atlantic Permanente Medical Group, Silver Spring, MD, United States; 2 New York Institute of Technology, College of Osteopathic Medicine, Jonesboro, AR, United States

**Keywords:** glioblastoma multiforme, IDH wild type GBM, molecular case study, rheumatoid arthritis, TERT promoter negative GBM, TNF- alpha inhibitor

## Abstract

TNF α inhibitors such as adalimumab are widely used for autoimmune diseases, yet their long-term impact on tumor development in genetically susceptible individuals remains incompletely defined. Glioblastoma (GBM) is an aggressive IDH wild-type tumor with recurrent molecular alterations; however, comprehensive genomic analyses of GBM arising in patients treated with TNF-α inhibitors are extremely limited. We examined the genomic features of a GBM developing after prolonged TNF-α inhibitor therapy to explore potential links between TNF-α blockade, and tumor evolution. Tumor tissue was analyzed using immunohistochemistry, targeted next-generation sequencing, and copy number analysis performed at two independent clinical laboratories. Genomic findings were interpreted in the context of TNF α pathway biology and tumor microenvironment interactions relevant to GBM progression. The tumor demonstrated GFAP and OLIG positivity, a Ki-67 index of 45%, and strong p53 expression (>90%). Genomic profiling revealed hallmark alterations of IDH wild-type GBM, including CDKN2A/B deletions, PTEN deletion, and a TP53 mutation. Additional findings included a KDM6A frameshift variant, an ATRX variant of uncertain significance, and loss of PDPK1. No TERT promoter mutation was detected, suggesting a potential alternative telomere maintenance mechanism. The combination of PTEN loss, TP53 mutation, and CDKN2A/B deletion is consistent with an aggressive molecular phenotype associated with immune evasion. This case highlights genomic features of an IDH−wild−type glioblastoma arising after prolonged TNF−α inhibitor exposure. While no causal inference can be made, this analysis identifies both canonical and atypical genomic alterations in a GBM arising after prolonged TNF-α targeted therapy. This temporal relationship provides a basis for studying possible convergence between TNF-α signaling and GBM-associated pathways, and underscores the importance of genomic risk stratification when considering TNF-α inhibitor therapy.

## Introduction

Tumor necrosis factor–alpha (TNF−α) inhibitors are widely used to treat autoimmune diseases, including rheumatoid arthritis (RA), psoriasis, and ulcerative colitis, because of their ability to suppress chronic inflammation and improve clinical outcomes (1–3). RA, which predominantly affects women (4) has a bimodal age distribution (5). While these biologics have transformed care, long−term modulation of TNF−α signaling raises concerns about altered immune surveillance and malignancy risk, particularly in older patients and those with potential predisposition (6). Guidance for pre−treatment identification of individuals at heightened oncologic risk remains limited.

Glioblastoma (GBM), the most aggressive primary brain tumor in adults, exhibits recurrent molecular alterations affecting cell−cycle control, tumor−suppressor pathways, and telomere maintenance. In IDH−wild−type GBM, frequent CDKN2A/B deletions, PTEN loss, and TP53 mutations are often accompanied by TERT promoter mutations (7, 8) enabling telomerase−dependent immortalization; in their absence, alternative lengthening of telomeres (ALT) may occur (9, 10). Emerging work also highlights replication−repair deficient subtypes with immunogenic features that may intersect with immune signaling axes (11).

TNF−α signaling plays context−dependent roles in cancer biology—capable of promoting or restraining tumor progression depending on microenvironmental cues (12, 13). In glioma models, TNF−α has been implicated in angiogenic factor upregulation and invasive behavior (14).

Clinical observations have reported central nervous system malignancies, including glioblastoma, in the setting of TNF−α blockade (15, 16), though systematic genomic characterization has been sparse.

Here, we present a comprehensive genomic analysis of an IDH−wild−type GBM that developed after prolonged exposure to TNF−α−targeted therapy, integrating clinical history with immunohistochemistry, targeted next−generation sequencing, and copy−number analysis performed across two independent laboratories.

## Methods and clinical course

Tumor tissue was analyzed using immunohistochemistry, targeted next−generation sequencing, and copy−number analysis performed at two independent CLIA−certified laboratories. The methylation−specific PCR, targeted NGS, and copy−number analyses were performed in a CLIA−certified clinical molecular diagnostic laboratory, using validated proprietary workflows.

The patient was a woman in her early 50s with a history of rheumatoid arthritis, asthma, gastroesophageal reflux disease, hyperlipidemia, anxiety, and depression. She initially presented with inflammatory polyarthritis involving the distal and proximal interphalangeal and metacarpophalangeal joints, associated with morning stiffness and functional limitation. Family history was notable for rheumatoid arthritis in a first−degree relative. Laboratory evaluation demonstrated a borderline elevation of rheumatoid factor, and radiographic evaluation of the hands was unremarkable.

Initial management included nonsteroidal anti−inflammatory therapy followed by corticosteroids, with symptomatic improvement. Disease−modifying antirheumatic treatment was initiated with methotrexate and folic acid, later adjusted due to hepatotoxicity. Sulfasalazine was subsequently added. Owing to persistent disease activity, biologic therapy with the TNF−α inhibitor adalimumab was introduced while methotrexate was continued at a reduced dose. The patient had received adalimumab at a dose of 40 mg every two weeks for approximately 4–4.5 months; the glioblastoma was diagnosed 9 months after treatment initiation and 5 months after discontinuation. Adalimumab was later discontinued due to gastrointestinal intolerance, and therapy was transitioned to tofacitinib (Janus kinase inhibitor) in combination with methotrexate.

In the following months, after initiation of targeted TNF-α inhibition therapy, the patient developed new−onset left upper extremity weakness. Magnetic resonance imaging of the brain revealed a right−sided mass involving the basal ganglia and thalamus. Stereotactic biopsy confirmed the diagnosis of glioblastoma multiforme.

The patient was treated with corticosteroids and temozolomide, with plans for chemoradiation. Her clinical course was complicated by an intracranial hemorrhage, leading to progressive neurologic decline. These included increased confusion and incontinence as a result of an intracranial bleed. Goals of care were transitioned to comfort−focused management, and the patient died several weeks later.

Immunohistochemical analysis demonstrated diffuse positivity for GFAP and OLIG, strong nuclear p53 expression in greater than 90% of tumor cells, and a Ki−67 proliferation index of approximately 45%. Targeted sequencing and copy−number analysis were performed to characterize the molecular features of the tumor and are presented in the following section.

## Results

### Molecular and genomic findings

Immunohistochemical analysis demonstrated diffuse tumor cell positivity for glial fibrillary acidic protein (GFAP) and OLIG, confirming glial lineage. Nuclear accumulation of p53 was observed in greater than 90% of tumor cells, and the Ki−67 proliferation index was approximately 45%, consistent with a highly proliferative neoplasm. Staining for cytokeratin AE1/AE3 and CD45−LCA was negative. IDH1 R132H mutation testing was negative, supporting classification as IDH−wild−type glioblastoma. These results were reviewed and confirmed, which also conducted additional molecular diagnostic tests:

- IDH1 R132H mutation was negative.
- ATRX expression was retained.
- Genetic and Molecular Diagnostic Results (**Table 1**).

**Table 1:** Genomic alterations identified by targeted NGS and copy−number analysis in the IDH−wild−type glioblastoma tumor specimen.

Gene/chromosomal location	Results	Notes/comments
MGMT promoter methylation	Positive CpG methylation detected in the MGMT promoter region.	Methylation index: 16, Methylation fraction: 0.961, Methylation score: 15.37
CDKN2A, CDKN2B	Homozygous/biallelic deletion	
KDM6A	Frameshift mutation	Mutation codon details (Ref, Alt) unavailable
PTEN	Homozygous/biallelic deletion	
TP53	Loss of Heterozygosity (LOH) with recurrent missense mutation	Mutation codon details (Ref, Alt) unavailable
PDPK1	Deletion	
ATRX	Missense mutation (uncertain significance)	Mutation codon details (Ref, Alt) unavailable
Chromosome 4	4p Distal loss	Position details unavailable
Chromosome 7	Gain of 7p and proximal 7q	Position details unavailable
Chromosome 9	Distal loss at 9p21.3	
Chromosome 10	Loss at 10q23.3	
Chromosome 11	11p Gain	
Chromosome 13	Interstitial 13q loss, distal 13q gain	
Chromosome 16	16p loss, proximal 16 q gain, distal 16 q loss	
Chromosome 17	Distal 17p loss	
Chromosome 18	Distal 18q loss	
Chromosome 19	19q Loss	
Chromosome 20	Distal 20q loss	
Chromosome 22	22q Loss	
Chromosome X	p arm Loss	

**Figure 1:**
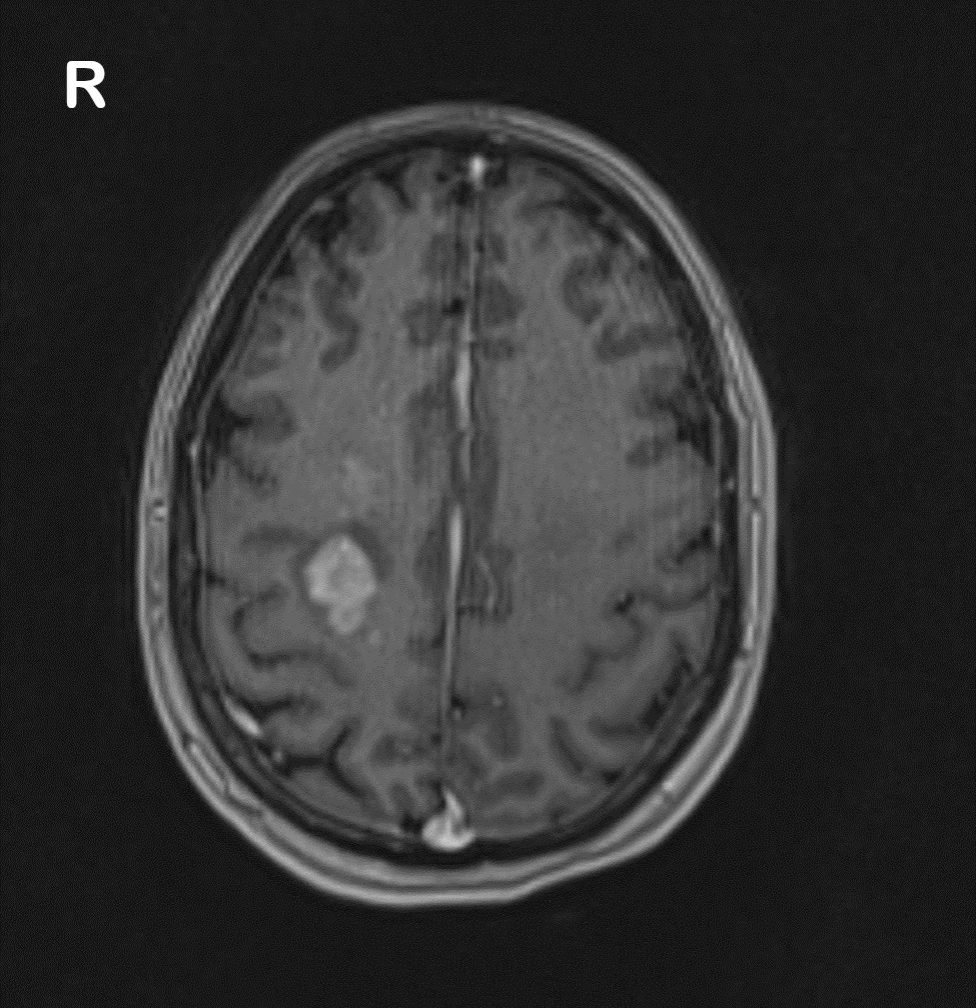
MRI of the brain demonstrating a right-sided mass involving the basal ganglia and thalamus.

MRI results further confirmed the diagnosis (**Figure 1**).

Targeted next−generation sequencing and copy−number analysis identified multiple genomic alterations characteristic of adult IDH−wild−type glioblastoma, along with additional atypical features. A consolidated summary of sequence variants, copy−number changes, and chromosomal alterations is provided in **Table 1**.

### Genetic alterations

Canonical alterations included homozygous deletions involving CDKN2A and CDKN2B, deletion of PTEN, and loss of heterozygosity affecting TP53 with a recurrent missense variant. These alterations are commonly associated with aggressive clinical behavior in IDH−wild−type glioblastomas. Copy−number analysis revealed whole and partial chromosomal gains and losses frequently observed in this tumor type, including gains involving chromosome 7 and losses involving chromosome 10.

Additional genomic findings included a frameshift variant involving KDM6A, loss of PDPK1, and a missense variant in ATRX classified as a variant of uncertain significance. ATRX protein expression was retained by immunohistochemistry. The KDM6A frameshift variant lacked sufficient annotation to define its precise genomic location, consistent with a likely loss−of−function event.

Notably, no mutation was detected in the promoter region of TERT, despite the presence of other hallmark genomic features of IDH−wild−type glioblastoma. MGMT promoter analysis demonstrated CpG island methylation.

Collectively, the molecular profile identified canonical high−risk alterations typical of IDH−wild−type glioblastoma alongside atypical features, including the absence of a TERT promoter mutation and the presence of noncanonical epigenetic and chromatin−associated variants. These findings establish a genomic framework for the subsequent discussion of tumor biology and potential interactions with TNF-α inhibition therapies.

## Discussion

This molecular profile illustrates both canonical drivers of IDH−wild−type glioblastoma and atypical features that may inform tumor evolution and immune interactions. Homozygous CDKN2A/B deletions, PTEN loss, and TP53 mutation collectively underpin dysregulated cell−cycle control, PI3K/AKT signaling, and genomic stress responses typical of aggressive GBM. The copy−number pattern—including 7p/7q gains and 10q loss—further aligns with adult IDH−wild−type disease biology. In contrast, the absence of a TERT promoter mutation raises the possibility of non−telomerase telomere maintenance, such as ALT, which has been described in subsets lacking canonical TERT activation (9, 10). Although TERT promoter mutations occur in the majority of IDH−wild−type glioblastomas, roughly 15–25% do not exhibit these alterations, suggesting that they rely on alternative telomere−maintenance pathways (17). Thus their absence in cases such as these warrant further investigation. The ATRX variant of uncertain significance, with retained protein expression, complicates this inference; nevertheless, chromatin remodeling perturbations can intersect with telomere dynamics and genomic stability. These observations reinforce that telomere maintenance in IDH−wild−type GBM is heterogeneous and may influence therapeutic vulnerability.

Additional alterations—KDM6A frameshift and PDPK1 loss—suggest epigenetic and signaling consequences beyond classical GBM hallmarks. Although not specific to glioma, KDM6A loss has been associated with an epigenetic reprogramming phenotype, proliferative advantage, and therapy resistance in other malignancies (18–20). The convergence of tumor−suppressor loss (PTEN, TP53) with potential chromatin and epigenetic dysregulation may contribute to immune−evasive behavior within the tumor microenvironment—an axis increasingly recognized in IDH−wild−type GBM, including replication−repair–deficient subtypes with immunologic features (11, 21).

Contextualizing these findings with TNF−α biology requires careful analysis and considerations. TNF−α signaling exerts dual, context−dependent roles in cancer—capable of promoting survival, invasion, and angiogenic factor expression in glioma models, while also mediating cytotoxic and immune−stimulatory effects under different conditions (12–14). Furthermore, TNF-alpha inhibitors have been shown to have complex interactions with tumor microenvironments, potentially reducing inflammation and tumor-promoting signaling pathways (22). Although the mechanistic basis for malignancy risk under TNF−α inhibition remains unresolved, epidemiologic signals have been reported for glioblastoma (15, 16, 23) and for other cancers, including nonmelanoma skin cancer and non−Hodgkin lymphoma (6). These observations justify prospective epidemiologic studies explicitly integrated with tumor and host genomics, leveraging next−generation sequencing (NGS) to delineate how TNF-α inhibition approach intersects with oncogenic signaling and telomere−maintenance networks. In this case, the temporal relationship with prolonged biologic therapy and the convergence of alterations affecting PTEN, TP53, chromatin regulation, and immune−associated pathways support a hypothesis−generating framework centered on mechanistic vulnerability.

Clinically, these data support genomic risk stratification and systematic tumor profiling in patients receiving long−term TNF-α inhibition therapy, especially when additional susceptibility is plausible (family history, clinical course). Prospective efforts linking pre−therapy baseline genomics, longitudinal exposure, and tumor molecular phenotypes at diagnosis will be essential to clarify whether and how TNF-α pathway modulation intersects with GBM evolution.

## Conclusion

This study describes a glioblastoma arising after prolonged TNF−α−targeted therapy and defines a genomic profile comprising both canonical high−risk alterations and atypical features, including absence of a TERT promoter mutation and chromatin−associated variants. While mechanistic causality cannot be inferred, the convergence of tumor−suppressor loss, epigenetic disruption, and immune−regulatory pathways highlights a biologically informative context for hypothesis generation. There is clearly a need for further systematic epidemiologic studies coupled with tumor and host genomic profiling to determine any level of association or otherwise between use of such therapies and genomic susceptibility to developing GBM. Leveraging next−generation sequencing can help clarify the molecular contexts in which TNF−α pathway modulation might intersect with glioblastoma development, providing a framework for future mechanistic and epidemiologic investigations. Such approaches can complement familial risk assessment and baseline screening strategies in patients considered for long−term treatment using such TNF-α inhibitors.

## Data Availability

The datasets presented in this article are not readily available because they derive from a single human participant and include genomic data subject to participant/patient anonymity and privacy considerations. Requests to access the datasets should be directed to the corresponding author.
